# Geo-Sensing-Based Analysis of Urban Heat Island in the Metropolitan Area of Merida, Mexico

**DOI:** 10.3390/s24196289

**Published:** 2024-09-28

**Authors:** Francisco A. Sánchez-Sánchez, Marisela Vega-De-Lille, Alejandro A. Castillo-Atoche, José T. López-Maldonado, Mayra Cruz-Fernandez, Enrique Camacho-Pérez, Juvenal Rodríguez-Reséndiz

**Affiliations:** 1 Facultad de Ingeniería, Universidad Autónoma de Yucatán, Mérida 97000, Mexico; a17014854@alumnos.uady.mx (F.A.S.-S.); marisela.vega@correo.uady.mx (M.V.-D.-L.); acastill@correo.uady.mx (A.A.C.-A.); 2 División de Tecnologías Industriales, Universidad Politécnica de Querétaro, El Marques 76240, Mexico; jose.lopez@upq.edu.mx (J.T.L.-M.); mayra.cruz@upq.edu.mx (M.C.-F.); 3 Red de Investigación OAC Optimización, Automatización y Control, El Marqués 76240, Mexico; 4 Facultad de Ingeniera, Universidad Autónoma de Queretaro, Querétaro 76010, Mexico

**Keywords:** urban heat island, geo-sensing, machine learning, remote sensing, image processing, computer vision

## Abstract

Urban Heat Islands are a major environmental and public health concern, causing temperature increase in urban areas. This study used satellite imagery and machine learning to analyze the spatial and temporal patterns of land surface temperature distribution in the Metropolitan Area of Merida (MAM), Mexico, from 2001 to 2021. The results show that land surface temperature has increased in the MAM over the study period, while the urban footprint has expanded. The study also found a high correlation (r> 0.8) between changes in land surface temperature and land cover classes (urbanization/deforestation). If the current urbanization trend continues, the difference between the land surface temperature of the MAM and its surroundings is expected to reach 3.12 °C ± 1.11 °C by the year 2030. Hence, the findings of this study suggest that the Urban Heat Island effect is a growing problem in the MAM and highlight the importance of satellite imagery and machine learning for monitoring and developing mitigation strategies.

## 1. Introduction

Urban Heat Islands (UHIs) are areas that experience higher temperatures than their surroundings due to deforestation and consequent urbanization. This effect can particularly affect tropical cities, which already experience a warm climate. Extreme air temperatures contribute directly to deaths from cardiovascular and respiratory diseases, as they lead to increased levels of ozone and other air pollutants [1]. In addition, people in areas affected by UHI are more prone to heat stroke (hyperthermia), a severe overheating of the body due to high temperatures. The population most affected by extreme temperatures are children, the elderly and people living in poverty and/or are homeless [2]. Moreover, days of intense heat cause an increase in energy demand through intensive use of ventilation systems and air conditioners, increasing the generation of greenhouse gases [3].

**Figure 1:**
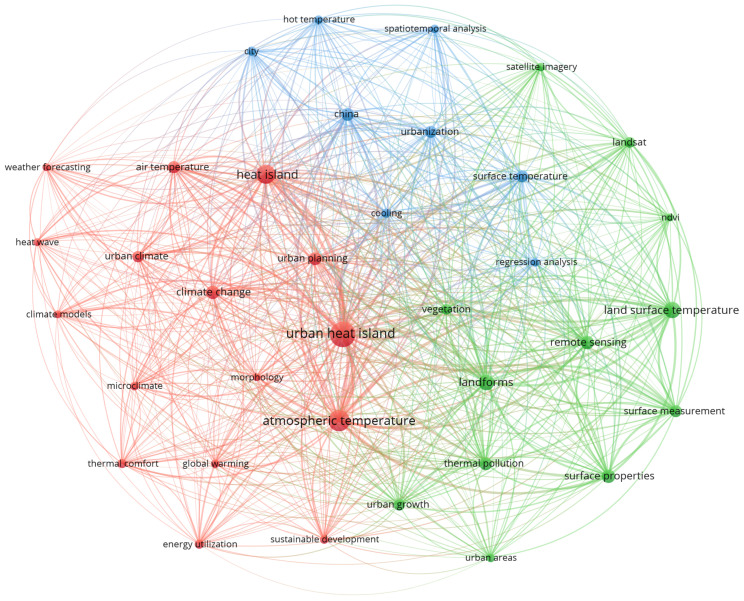
Analyzing the interrelationships between keywords and research trends in publications retrieved from Scopus.

In order to better understand the current research in this field, UHI was used in a keyword search of the literature from the last five years (2019–2023). After selection, a total of 1271 publications that met the requirements were selected. The VOSviewer program was used for analysis, and a total of 45 keywords were included in the visualization. The outcomes of the analysis of highlighted words are presented in Figure 1. The figure exhibits three distinct color clusters, namely, red, blue, and green. The analysis reveals that heat island and Urban Heat Island are the terms that occur the most frequently, highlighting the central focus of UHI studies. Keywords such as atmospheric temperature, landforms, and surface temperature reflect the emphasis on understanding the physical and environmental factors influencing UHI. The presence of remote sensing, Landsat, and spatiotemporal analysis demonstrates the extensive use of advanced technologies in these studies. Furthermore, regression analysis is prominent, highlighting its role in quantitatively assessing the relationships between UHI and various influencing factors. The focus on urbanization, urban planning, and climate change indicates a growing interest in the relationship between UHI and broader urban and environmental challenges.

Research on the UHI phenomenon focuses on understanding its progression, which can be assessed using sensors at stationary meteorological stations and dedicated platforms such as tall towers, radiosondes, or airplanes. However, configuring these devices is laborious and expensive, limiting their accessibility to a few prominent locations around the world [4]. However, satellite remote sensing data offer reliable and replicable measurements, enabling the examination of urban thermal conditions across various geographical and temporal dimensions, from small-scale to large-scale and from daily to yearly. Furthermore, satellite data can be utilized to accurately forecast Urban Heat Island (UHI) patterns. The satellite-based exploration of UHIs began in 1979 [5], marking a significant milestone in understanding urban thermal dynamics. In particular, three main satellite sensors, Landsat, MODIS, and ASTER, stand out for their contributions to UHI research. Landsat has been extensively employed, encompassing four generations of sensors [6]. Its longevity, accessibility, and moderate spatial resolution have made it a cornerstone in UHI investigations. MODIS has global coverage; its data are used to research large study areas due to spatial resolution [7]. Although ASTER imagery has historically been limited due to cost constraints, its availability since 2016 has opened new avenues for UHI research, especially with its fine spatial resolution and diurnal temperature variation analysis capabilities [8].

One of the main advantages of using satellites can also be seen as a disadvantage: the large amount of data obtained is difficult to process due to storage limitations or lack of access to high-end computing equipment. Google Earth Engine (GEE) facilitates access to petabytes of data from satellites such as Landsat [9]. In addition, it allows the use of its servers for cloud processing, eliminating one of the significant disadvantages of using satellite data.

As examples of the use of satellite information, we can mention the analysis conducted by [10], who investigated UHIs in the Changsha-Zhuzhou-Xiangtan (CZT) region from 2000 to 2017 using Landsat data. The results revealed the intensity of the UHI peaks in summer, varying between cities. Furthermore, the UHI centers were moving closer, indicating urban aggregation, with differing trends observed in the surrounding areas and urban cores. Land cover change, particularly the increase in impervious surfaces, was identified as the primary driver of UHI variations, with industrial activity also influencing the observed patterns.

Ref. [11] examined the Urban Heat Island (UHI) trends in Delhi, considering aerosol levels and land-cover changes. They utilized data from MODIS for their analysis. The study found that night-time temperatures in Delhi consistently remained higher than in surrounding areas throughout the year, with the least difference observed during the monsoon season and the highest difference in March. During the daytime, occasional cooling over Delhi was noted, particularly in May–June and October–December. The analysis revealed a negative correlation between UHI intensity and aerosol levels, with land-cover changes also significantly shaping UHI patterns.

In the study conducted by [12], the Urban Heat Islands (UHIs) in Tehran and its suburbs were compared using day/night data from MODIS and Landsat. Higher UHI values were found in downtown Tehran and suburban areas at night, with the temperature difference increasing in Tehran and western suburbs at night but decreasing during the day. Analysis using Moran’s I and the Getis index revealed consistent patterns, with a seasonal and annual UHI difference of 5 °C between Tehran and its suburbs. Elevated temperatures were observed during warm months, and lower NDVI values indicated reduced vegetation from June to February. Urban areas with sparse vegetation exhibited higher UHI values at night than suburbs, suggesting an expansion of UHI beyond city borders.

In the tropical city of Merida, Mexico, it has been reported that the land surface temperature (LST) can increase between 2.36 and 3.94 °C after deforestation and the consequent urbanization of defined areas [13]. Likewise, the mean annual air temperature has progressively increased since the 1960s. In particular, this increase has accelerated in the last two decades, as urban growth has expanded exponentially [3]. Analysis of the behavior and evolution of the Urban Heat Island in Merida, Mexico, may improve urban planning and protect the inhabitants from environmental risks caused by this phenomenon.

The present research makes three significant contributions to understanding Urban Heat Islands (UHIs) in Merida, Yucatan, Mexico. First, it provides a comprehensive analysis of UHI dynamics over 21 years using satellite data, offering a long-term perspective on urbanization’s impact on local climate. Second, it integrates advanced processing techniques via Google Earth Engine, enhancing the accuracy of UHI assessments in tropical environments. Third, the research establishes a clear correlation between urban expansion and UHI intensification, offering valuable insights for urban planning and environmental management.

This work is structured as follows. Section 2 details the study area in Merida, Yucatan, and outlines the data sources and methodologies employed, including the processing of MODIS and Landsat imagery with Google Earth Engine. Section 3 presents the results, focusing on the spatio-temporal analysis of UHI and its correlation with urbanization trends, and Section 4 discusses the implications of our findings compared to similar studies. Finally, Section 5 concludes the study by summarizing the main contributions.

## 2. Materials and Methods

### 2.1. Study Area

The study area is the Metropolitan Area of Merida (MAM), Yucatan, Mexico. According to the Mexican National Institute of Statistics and Geography (INEGI, for its acronym in Spanish), the MAM comprises eleven municipalities. However, only three of them are considered central municipalities because they share an inter-municipal conurbation: Kanasn, Merida and Umán [14], and thus are the ones included in this study. The central municipalities encompass the towns of Kanasín, Merida, Caucel, Chablekal, Cholul, Komchén, San José Tzal, Leona Vicario, and Umán, which cover an area of approximately 1334 km^2^ and contain more than 1 million inhabitants (approximately 45% of the population of the state of Yucatan). The municipalities’ vector data were obtained from INEGI’s geostatistical framework 2021 [15].

**Figure 2:**
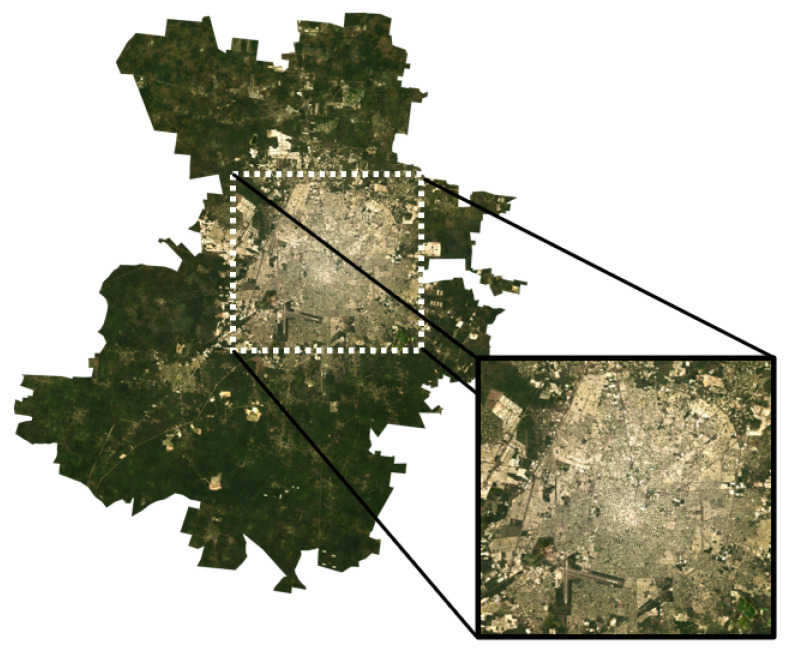
Study area corresponding to the MAM, Yucatan, Mexico.

Moreover, to accurately evaluate the effect of UHI in MAM, within the three municipalities chosen for this study, a main area was defined further as shown in Figure 2. The remaining surface was established as the UHI surroundings.

The Metropolitan Area of Mérida (MAM) was selected for its rapid urban expansion and availability of high-quality satellite data. As a representative of medium-sized cities in tropical climates undergoing significant urbanization, the study area includes diverse land cover types—urban, peri-urban, and rural—offering a thorough analysis of UHI impacts across the region.

### 2.2. Land Surface Temperature

The LST evaluated in this study was obtained from the *Terra Moderate Resolution Imaging Spectroradiometer (MODIS) Land Surface Temperature/Emissivity Daily (MOD11A1) Version 6.1* database. The database has a spatial resolution of 1000 m and contains information from 24 February 2000 to the present. The LST derived from MODIS images is obtained on a daily basis [16]. The bands downloaded and the data they contain are presented in Table 1.

The bands information was downloaded in two formats using Earth Engine Code Editor (EECE): CVS and TIF. The CVS files were processed using Pandas library, Matplotlib, and NumPy in Python. In contrast, TIF files were converted to PNG with QGIS software to be processed using the OpenCV library in Python.

From the recovered data, 21 LST maps (one for each year from 2001 to 2021) were created after calculating pixel-by-pixel the mean annual LST, averaging day and night readings. Likewise, histogram graphs were generated to further evaluate the evolution of the LST in the MAM.

### 2.3. Urban Footprint Classification

For classification of the urban footprint in the study area, surface reflectance (SR) bands were obtained from the *USGS Landsat 8 Level 2, Collection 2, Tier 1* database. The database has a spatial resolution of 30 m and contains information from 18 March 2013 to the present. Each image is taken approximately every two weeks [17]. Table 2 shows the downloaded bands and the data they contain.

The band information was downloaded in TIF format using the EECE. In order to reduce the effects caused by cloud cover, the available images obtained per year were averaged. Afterwards, the images were converted to PNG format with QGIS software to be processed using the OpenCV library in Python.

To mitigate cloud cover-derived inaccuracies, all available yearly images were averaged. In the MOD11A1.061 Terra Land Surface Temperature and Emissivity Daily Global 1 km dataset, there are around 360 images per year, and by averaging them, it is expected that the yearly average will be less prone to fluctuations. Similarly, for the USGS Landsat 7 Level 2, Collection 2, Tier 1 dataset, which contains approximately 20 images per year, the same approach was applied. Both the MODIS and Landsat Tier 1 datasets already process cloud cover to some extent, so beyond averaging the images, no further processing was necessary. Data normalization was not required, as the datasets already provided the information in a format ready for analysis.

**Figure 3:**
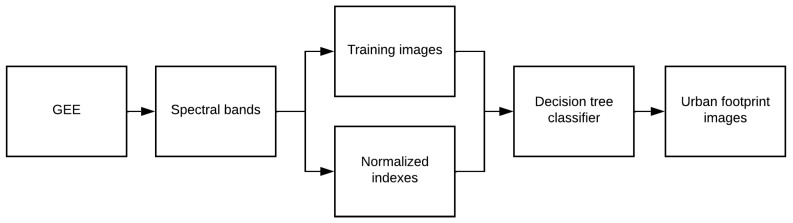
Flowchart of the urban footprint classification process.

From the recovered data, 8 urban footprint maps (one for each year from 2014 to 2021) were created after pixel-by-pixel classification. This process was performed following the decision tree classifier shown in Figure 3. The decision tree classifier was selected due to hardware constraints, offering an optimal balance between speed and accuracy for processing large datasets (9 million pixels per image).

For the determination of the normalized indexes, Equations (1) and (2) are applied [18,19]:

(1) $$\begin{array}{cc} \mathrm{NDVI} & =\frac{\mathrm{SR}\_B5-\mathrm{SR}\_B4}{\mathrm{SR}\_B5+\mathrm{SR}\_B4} \end{array}$$

(2) $$\begin{array}{cc} \mathrm{NDWI} & =\frac{\mathrm{SR}\_B3-\mathrm{SR}\_B5}{\mathrm{SR}\_B3+\mathrm{SR}\_B5} \end{array}$$

Here, NDVI corresponds to the Normalized Difference Vegetation Index and NDWI to the Normalized Difference Water Index. Subsequently, the training process consists of assigning an identifier (ID) to each pixel according to the classes defined in Table 3.

Vegetation and water proved challenging to differentiate in swampy areas, such as mangroves, where both water and vegetation indexes were positive. To resolve this, water was prioritized due to fewer water pixels, yielding better results with the machine learning algorithm. Additionally, distinguishing between built-up areas and vegetation was difficult, as barren ground and dead vegetation provided similar data. To address this, the range for positive results in both indexes was reduced, leading to fewer but more accurate data points. With access to all the spectral bands, the machine learning algorithm was expected to find a pattern that would improve the classification of missing data points.

Once classified images (urban footprint maps) were obtained, the NumPy and Pandas libraries were used to calculate statistics to evaluate deforestation/urbanization during the studied period in the MAM.

#### 2.3.1. Urban Footprint Validation

To validate the results obtained from the decision tree classifier, a confusion matrix and the corresponding Kappa index were used for comparison with a reliable reference information [20]. The confusion matrix and the Kappa index formula are shown in Table 4 and Equation (3), respectively.

(3) $$\begin{array}{cc} K & =\frac{N\sum_{i=1}^{n}m_{i,i}-\sum_{i=1}^{n}\left(G_{i}\times C_{i}\right)}{N^{2}-\sum_{i=1}^{n}\left(G_{i}\times C_{i}\right)} \end{array}$$

where *i* is the class number, *N* is the total number of values compared, $m_{i,i}$ is the number of values belonging to class *i* that were classified as class *i*, $C_{i}$ is the total number of predicted values belonging to class *i*, and $G_{i}$ is the total number of reference values belonging to class *i* [21]. The interpretation of the Kappa index values is shown in Table 5.

The reference data used as a reliable source were taken from the *ESA WorldCover 10m v100* database, which provides a global land cover map of the year 2020 with a resolution of 10 m derived from satellites Sentinel-1 and Sentinel-2 data, encompassing 11 land cover classifications. This database is considered a reliable source because it was previously statistically validated using another multi-use global land cover validation dataset, the *CGLS-LC 10m v100* [23].

Since the database only contains information from 2020, the urban footprints maps considered for the validation are those corresponding to the closest years, that is, 2019, 2020, and 2021.

### 2.4. Urban Heat Island Analysis

To evaluate the correlation between LST and the urban footprint (land cover classes), the Pearson coefficient shown in Equation (4) was used:

(4) $$\begin{array}{c} r=\frac{\sum_{i=1}^{N}\left(x_{i}-\bar{x}\right)\left(y_{i}-\bar{y}\right)}{\sqrt{\sum_{i=1}^{N}{\left(x_{i}-\bar{x}\right)}^{2}\sum_{i=1}^{N}{\left(y_{i}-\bar{y}\right)}^{2}}} \end{array}$$

where $x_{i}$ are the LST values, $\bar{x}$ is the mean of the LST, $y_{i}$ are the values of the corresponding land cover class area, $\bar{y}$ is the mean of the corresponding land cover class area. To perform the calculations, the total hectares of the MAM land cover classes identified during classification were determined (variable *y* in Equation (4)) for each available year (from 2014 to 2021). Afterwards, these values were contrasted with the average pixel-by-pixel LST annual means from 2014–2021 (variable *x* in Equation (4)).

In addition, the average of the pixel-by-pixel LST annual means of each studied year (from 2001 to 2021) was calculated for the MAM as well as for its surroundings (see Figure 2).

Afterwards, the difference between both values was plotted to be able to fit a linear regression curve for forecasting purposes.

## 3. Results

### 3.1. Land Surface Temperature Results

**Figure 4:**
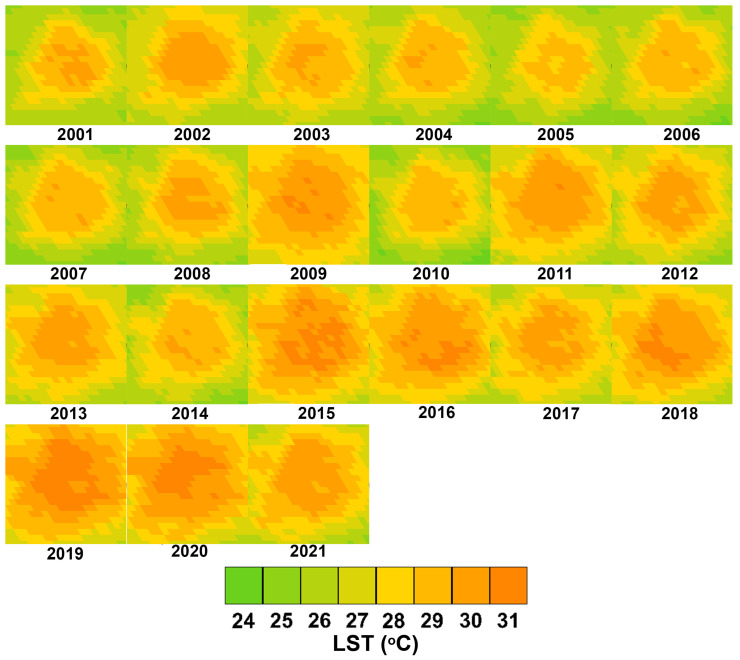
LST maps of the MAM.

The 21 LST maps obtained for the study area from 2001 to 2021 are shown in Figure 4. From the initial visual analysis, the expansion and augmentation of yearly mean LST above 30 °C throughout the MAM become evident, suggesting the presence of the UHI effect. It is considered important to remark that the year 2009 shows an atypical significant increase in LST. This was associated to an unusual number of small dry vegetation fires (common during dry season) observed in that year in the MAM [24].

**Figure 5:**
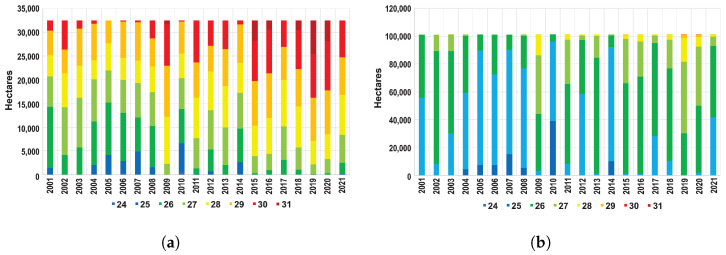
LST distribution in (**a**) the MAM and (**b**) the MAM surroundings.

To further evaluate the evolution of the LST in the MAM, Figure 5a shows a histogram showing the distribution of the LST per hectare per year.

Temperatures between 29 °C and 31 °C can be seen to become more frequent, particularly from 2015, accounting for approximately 38.7% of the total surface area of the MAM in 2021, compared to 17.5% in 2001. Moreover, Figure 5b shows the LST distribution histogram corresponding to the area defined as surroundings in Section 2.1. Although LST in this area also shows a steady increase towards higher values, this increase might be mainly associated to its proximity to the MAM. However, the majority of LST values observed remain under 29 °C (over 61.3% in 2021), demonstrating the effect of the UHI in the MAM.

**Figure 6:**
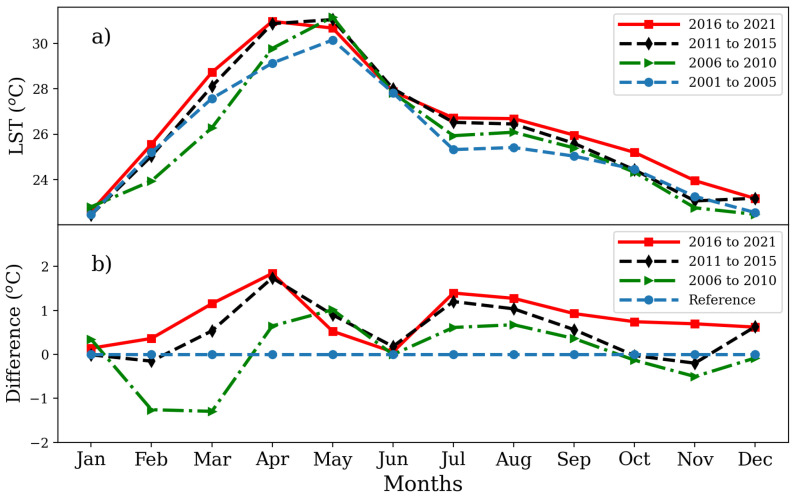
Monthly variation in (**a**) average LST of selected year intervals and (**b**) their difference with the interval 2001–2005 as reference value.

Finally, an analysis was conducted considering the LST variation per month. The approach consisted in classifying the available data in five-year intervals from 2001 to 2015 (i.e., 2001–2005, 2006–2010, and 2011–2015) and six-year intervals from 2016 to 2021, and calculating the average LST per month as shown in Figure 6a. The months with the highest LST were April, May, and June, and those with the lowest were November, December, and January. Particularly, the highest monthly LST readings obtained were 33.46 °C, 33.15 °C, and 32.28 °C for May 2011, June 2020, and May 2019, respectively. Likewise, the lowest LST were 20.1 °C, 20.54 °C, and 20.77 °C in January 2003, January 2018, and December 2010, respectively.

Furthermore, Figure 6b shows the LST difference between the considered intervals, with the first group taken as a reference (2001–2005). Particularly, for the last interval (2016–2021), the LST difference stood always above the reference line, with an increase greater than 1 °C in the months of April, July, and August.

### 3.2. Urban Footprint Results

For validation of the urban footprint maps generated, the Kappa index was evaluated as described in Section 2.3.1. The results obtained are shown in Table 6. According to Table 5, the level of agreement achieved is *substantial* (0.8–0.9 Kappa values) for the evaluated years (2019–2021), which represents overall performance over 94%. Therefore, the proposed supervised classification was considered reliable for evaluation of the urban footprint of the MAM. It is important to note that of the three classes evaluated (vegetation, built-up, and water), the water class was the one with the lowest individual precision, due to the fact that the study region does not contain any large water bodies and therefore only a few training data points were available.

**Figure 7:**
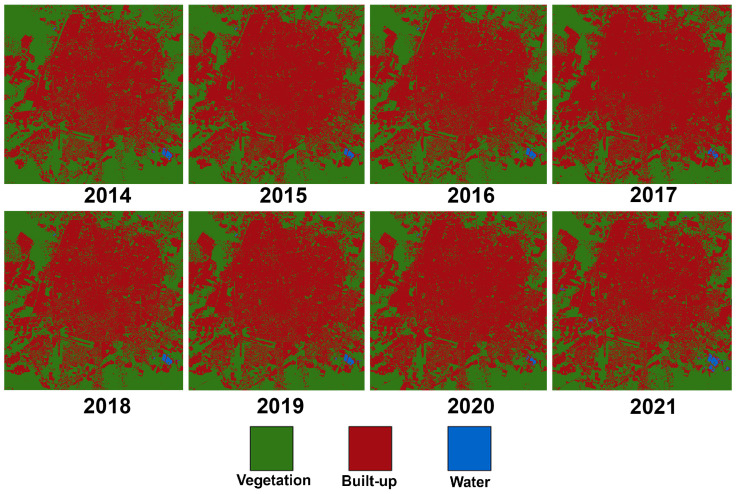
Urban footprint maps of the MAM.

The eight urban footprint maps obtained for the study area from 2014 to 2021 are shown in Figure 7. From the initial visual analysis, the expansion of the urban footprint and the associated decrease in vegetation can be observed.

Furthermore, the land cover area in hectares (ha) for the three evaluated classes (vegetation, built-up and water) from 2014 to 2021 is presented in Table 7. The results show that the built-up class occupies more than half of the total area, going from 51.32% in 2014 up to 59.68% in 2021. Consequently, there is a significant reduction of 17.65% of the vegetation class in the same period.

On the other hand, it was noticed that the year 2017 presented higher values of the built-up class than the following years, which is not compatible with the develop of an urban area. After further analysis, it was determined that cloud interference in that particular year affected the values calculated by the NDVI and the NDWI, leading to points belonging to the vegetation class being classified as built-up; hence, the data obtained for the urban footprint classification for 2017 were considered outliers. It is important to note that, for LST, satellite readings were obtained every two days, in contrast to every two weeks for the urban footprint. Therefore, cloud interference was not an affecting factor for the calculations of LST for the year 2017.

### 3.3. Urban Heat Island Analysis Results

Pearson’s correlation coefficients (Equation (4)) between LST and the two main land cover classes identified (vegetation and built-up) were calculated for the MAM using data from 2014 to 2021 (see Section 2.4), without considering the outlier year 2017. The results are shown in Table 8. The correlation between the vegetation class and LST is negative, while the correlation between the built-up class and LST is positive, both showing high agreement over 80%. The previous results clearly indicate, as expected, how LST increases with urbanization and the corresponding deforestation.

**Figure 8:**
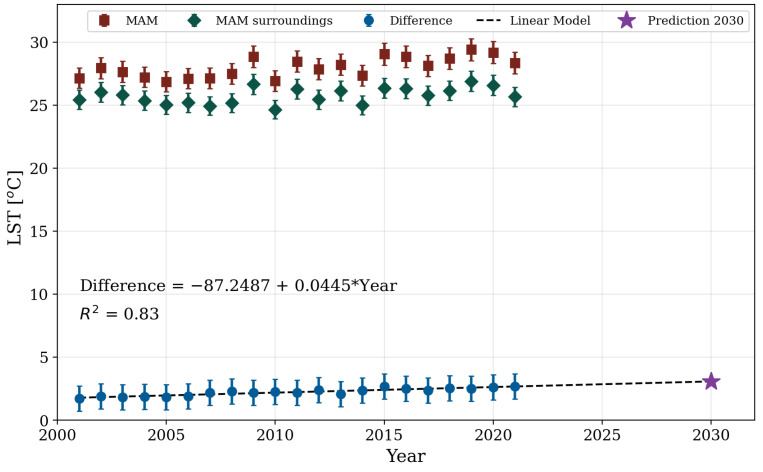
Average LST difference between the MAM and its surroundings from 2001 to 2021.

Likewise, Figure 8 shows the linear trend curve fitted to the data points obtained from the difference between the average LST of the MAM and the average LST of its surroundings (see Section 2.4) for each studied year (2001–2021). Additionally, the average values for the MAM and the MAM surroundings are also included. If the current trend continues, the temperature difference between the MAM and its surroundings is projected to reach 3.12 °C ± 1.11 °C by the year 2030. This value was obtained using Equation (5), which resulted from the fitted linear model (R^2^ = 0.83). To estimate the error, the RMSE was calculated based on the residual difference between the MAM temperature and that of its surroundings, yielding a value equal to 1.11 °C:

(5) $$\begin{array}{c} Difference=-87.2487+0.0445\times Year \end{array}$$

## 4. Discussion

Compared with other studies on Urban Heat Islands (UHIs), our findings align with the general consensus that urbanization contributes significantly to the increase in surface temperatures. A study by de Faria et al. on the UHI effect in Rio de Janeiro reported a similar pattern, demonstrating an increase in land surface temperature over time in urban areas [25]. When comparing our results with those of Al-Saadi et al., we observed similar seasonal trends in the intensity of UHI, with both studies showing increased UHI during periods of lower vegetation cover [26]. Agnieszka et al. also used Landsat data and in situ measurements to study the effect of heat island on urban heat [27]. They observed a mean annual UHI intensity of 1.0 °C in Pozna, with a higher intensity during warmer months and LST anomalies ranging from 3.4 °C in urban areas to -3.1 °C in forests. Similarly, we found notable spatial variability in the intensity of the UHI, which corroborated their findings on the influence of land use on UHI. The results presented by Kamal et al. align with our results, emphasizing the critical role of Urban Heat Islands in surface temperature variations [28]. They used the Google Earth Engine (GEE) platform, machine learning algorithms, and remote sensing data to assess LST, NDVI, and NDBI indices; a strong negative correlation between LST and NDVI, and a positive correlation between LST and NDBI were observed.

The correlation found in this study between urban expansion and LST (*r* > 0.8) is consistent with the values reported in other regions, such as the strong positive correlation (*r* = 0.988) between built-up areas and LST observed by Agrawal et al. [29]. Both studies suggest that urbanization is a significant driver of LST increases. Our study observed a rise in urban coverage from 51.32% in 2014 to 59.68% in 2021, while Waleed and Al-Bakri [30] reported a more dramatic increase from 17% to 53.2% in built-up areas over a longer period (2004–2021). Both trends correlate with significant increases in LST, further demonstrating how urban expansion accelerates UHI effects. This gradual but significant rise highlights the progressive impact of urbanization on thermal conditions, a trend also seen in other studies but with varying magnitudes depending on regional differences.

Our findings principally analyzed two different land cover types, where vegetation plays a critical role in reducing LST, while built-up areas contribute to its rise. Other studies considered additional contributors. In [31,32], it was shown that urbanization and industrial activities intensify Urban Heat Island (UHI) effects by altering land cover, increasing surface temperature, and reducing humidity. Further studies, such as [33,34], demonstrated that in addition to urbanization, vehicular emissions exacerbate UHI effects, negatively impacting local air quality and health.

While this study employs a Decision Tree model for the classification of cover types to analyze the Urban Heat Island, various studies highlight the effectiveness of other machine learning algorithms in predicting land surface temperatures and assessing urban heat vulnerability. For example, algorithms used for this purpose include Deep Neural Networks [35,36], Extreme Gradient Boosting [37], Random Forest [38,39], Support Vector Machines [40], and Bayesian Networks [41].

The study we presented is consistent with the findings of other researchers, reinforcing the critical role of urbanization in Urban Heat Island (UHI) formation and highlighting the importance of sustainable urban planning to mitigate heat stress in urban areas.

## 5. Conclusions

The average annual LST (2001–2021) and the urban footprint (2014–2021) of the MAM were obtained. The results show that LST has increased in the MAM over the study period, while the urban footprint has expanded, confirming the presence of the UHI phenomenon.

In particular, LST readings between 29 °C and 31 °C became steadily more frequent throughout the studied period, accounting for approximately 38.7% of the total surface area of the MAM in 2021, compared to 17.5% in 2001.

On the other hand, the built-up land cover class from the urban footprint increased, from covering 51.32% of the total area of the MAM in 2014 to 59.68% in 2021, correlating to a significant reduction of 17.65% of the vegetation land cover class.

The study found a high correlation (r> 0.8) between changes in LST and land cover classes (urbanization/deforestation). Hence, if the current urbanization trend continues, it is estimated that the difference between the LST of the MAM and its surroundings is expected to reach 3.12 °C ± 1.11 °C by the year 2030. Therefore, it is suggested that a public strategy for sustainable urban planning be implemented that incorporates elements that mitigate UHI, such as ecological corridors, green roofs, the use of reflective materials, and the development of green areas. These strategies should be applied in an integrated manner and in conjunction with the community and local authorities to achieve the effective mitigation of UHIs in the MAM.

## Figures and Tables

**Table 1:** Bands obtained from the *MOD11A1.061* database.

Band	Time Interval	Description
LST_Day_1km	1 January 2001–31 December 2021	Daytime LST
LST_Night_1km	1 January 2001–31 December 2021	Nighttime LST

**Table 2:** Bands obtained from the *USGS Landsat 8* database.

Band 4	Time Interval	Description	Wavelength (μm)
SR_B1 4	1 January 2014–31 December 2021	Ultra blue surface reflectance	0.435–0.451
SR_B2 4	1 January 2014–31 December 2021	Blue surface reflectance	0.452–0.512
SR_B3 4	1 January 2014–31 December 2021	Green surface reflectance	0.533–0.590
SR_B4 4	1 January 2014–31 December 2021	Red surface reflectance	0.636–0.673
SR_B5 4	1 January 2014–31 December 2021	Near-infrared surface reflectance	0.851–0.879
SR_B6 4	1 January 2014–31 December 2021	Short-wave infrared surface reflectance 1	1.566–1.651
SR_B7 4	1 January 2014–31 December 2021	Short-wave infrared surface reflectance 2	2.107–2.294

**Table 3:** Urban footprint class intervals and respective indexes.

ID	Classification	Index	Interval	Description
1	Vegetation	NDVI	0.4–1	Trees, shrubs, green grass, healthy vegetation
2	Built-up	NDVI	0.000001–0.399999	Concrete, pavement or other impervious material, sand, exposed rock, or exposed earth
3	Water	NDWI	0.1–1	Water body

**Table 4:** Confusion matrix for urban footprint validation [21].

Predicted Data (i)	Highly Reliable Reference Data (j)								
ID	Trees	Shrubland	Grassland	Cropland	Built-Up	Barren/Sparse Vegetation	Open Water	Herbaceous Wetland	
	Vegetation				Built-Up		Water		Total
Vegetation	$m_{1,1}$				$m_{1,2}$		$m_{1,3}$		$C_{1}$
Built-up	$m_{2,1}$				$m_{2,2}$		$m_{2,3}$		$C_{2}$
Water	$m_{3,1}$				$m_{3,2}$		$m_{3,3}$		$C_{3}$
Total	$G_{1}$				$G_{2}$		$G_{3}$		$\sum_{i=1}^{3}m_{i,i}$

**Table 5:** Interpretation of Kappa index values [22].

Kappa Value	Level of Agreement	Percentage of Valid Data
0–0.2	No agreement	0–4%
0.21–0.39	Slight	4–15%
0.4–0.59	Fair	15–35%
0.6–0.79	Moderate	35–63%
0.8–0.9	Substantial	64–81%
>0.9	Near Perfect	82–100%

**Table 6:** Urban footprint maps validation results.

Year	Kappa Index	Overall Accuracy	Class	Commission Errors	Omission Errors	Producer’s Accuracy	User Accuracy
2019	0.813	94.24%	Vegetation	2.17%	4.79%	95.21%	97.83%
			Built-up	20.68%	10.22%	89.78%	79.32%
			Water	6.84%	41.73%	58.27%	93.16%
2020	0.808	94.27%	Vegetation	1.58%	5.32%	94.68%	98.42%
			Built-up	21.98%	7.32%	92.68%	78.02%
			Water	7.37%	60.67%	39.33%	92.63%
2021	0.811	94.34%	Vegetation	2.07%	4.76%	95.24%	97.93%
			Built-up	20.31%	9.78%	90.22%	79.69%
			Water	50.37%	42.27%	57.73%	49.63%

**Table 7:** Land cover area in ha of the MAM from 2014 to 2021.

Year	Vegetation Class (ha)	Built-Up Class (ha)	Water Class (ha)
2014	17,133.7	18,138.9	69.2
2015	13,867.0	21,411.6	63.2
2016	15,260.0	20,007.2	74.6
2018	15,620.3	19,648.2	73.3
2019	13,875.6	21,393.6	72.6
2020	13,115.0	22,188.5	38.3
2021	14,110.4	21,092.1	139.2

**Table 8:** Pearson correlation between LST and land cover classes in the MAM.

Land Cover Class	*r*
Vegetation	−0.8180
Built-up	0.8234

## Data Availability

The database is available upon request to the authors.

## Abbreviations

The following abbreviations are used in this manuscript:

GEE	Google Earth Engine
MAM	Metropolitan Area of Merida
EECE	Earth Engine Code Editor
NDVI	Normalized Difference Vegetation Index
NDWI	Normalized Difference Water Index
LST	Land Surface Temperature
SR	Surface Reflectance
UHI	Urban Heat Island
RMSE	Root Mean Squared Error
